# Anti-cholinergic drug burden in patients with dementia increases after hospital admission: a multicentre cross-sectional study

**DOI:** 10.1186/s12877-022-03235-9

**Published:** 2022-10-06

**Authors:** Annabelle Hook, Jessica L. Randall, Carla M. Grubb, Natalie Ellis, Jack Wellington, Aayushi Hemmad, Agisilaos Zerdelis, Andrew R. D. Winnett, Benjamin D. W. Geers, Bethany Sykes, Charlotte N. Auty, Cecilia Vinchenzo, Christiane E. Thorburn, Daniella Asogbon, Emily Granger, Heather Boagey, Juliet Raphael, Kajal Patel, Kartik Bhargava, Mary-Kate M. Dolley, Matthew J. Maden, Mehdin M. Shah, Qao M. Lee, Ratnaraj Vaidya, Simran Sehdev, Sneha Barai, Sophie Roche, Uzair Khalid, David A. Codling, Judith R. Harrison

**Affiliations:** 1 Cardiff University School of Medicine, Neuadd Meirionnydd, Cardiff, CF14 4YS UK; 2 Great Western Hospital, Marlborough Road, Swindon, SN3 6BB UK; 3 Betsi Cadwaladr University Health Board, Bangor, LL57 2PW UK; 4 Withybush Hospital, Fishguard Road, Haverfordwest, SA61 2PZ UK; 5 The Medical School, Newcastle University, Framlington Place, Newcastle upon Tyne, NE2 4HH UK; 6 The James Cook University Hospital, Marton Road, Middlesbrough, TS4 3BW UK; 7 University Hospital of Wales, Heath Park Way, Cardiff, CF14 4XW UK; 8 Whipps Cross University Hospital, Whipps Cross Road, Leytonstone, London, EN11 1NR UK; 9 School of Medicine, University of Manchester, Manchester, M13 9PL UK; 10 University of Exeter Medical School, Heavitree Road, Exeter, EX1 2LU UK; 11 Queen’s Medical Centre Nottingham, Clifton Boulevard, Derby Road, Nottingham, NG7 2UH UK; 12 Lancaster Medical School, Faculty of Health and Medicine, Lancaster University, Furness Building, Lancaster, LA1 4YG UK; 13 Bristol Medical School, University of Bristol, First Floor, 5 Tyndall Avenue, Bristol, BS8 1UD UK; 14 Birmingham Medical School, College of Medical and Dental Sciences, University of Birmingham, Edgbaston, Birmingham, B15 2TT UK; 15 University Hospitals of Morecambe Bay NHS Foundation Trust, Burton Road, Kendal, LA9 7RG UK; 16 Lancashire Teaching Hospitals NHS Foundation Trust, Sharoe Green Lane, Fulwood, Preston, Lancashire PR2 9HT UK; 17 Medical Sciences Division, University of Oxford, John Radcliffe Hospital, Headley Way, Oxford, OX3 9DU UK; 18 Norwich Medical School, University of East Anglia, Norwich Research Park, Norwich, Norfolk NR4 7TJ UK; 19 Peninsula Medical School, The Faculty of Medicine and Dentistry, The John Bull Building, Plymouth Science Park, Research Way, Plymouth, PL6 8BU UK; 20 Bart’s and The London School of Medicine and Dentistry, Queen Mary’s University of London, Garrod Building, Turner Street, Whitechapel, London, E1 2AD UK; 21 Faculty of Medicine, University of Southampton, Building 85, Life Sciences Building, Highfield Campus, Southampton, SO17 1BJ UK; 22 School of Clinical Medicine, University of Cambridge, Box 111, Cambridge Biomedical Campus, Cambridge, CB2 0SP UK; 23 Peterborough City Hospital, Edith Cavell Campus, Bretton Gate, Peterborough, PE3 9GZ UK; 24 University College London Medical School, 74 Huntley St, Bloomsbury, London, WC1E 6DE UK; 25 St George’s Hospital, Blackshaw Road, Tooting, London, SW17 0QT UK; 26 King’s College London, Institute of Psychiatry, Psychology and Neuroscience, 16 De Crespigny Park, London, SE5 8AF UK; 27 Biomedical Research Building Campus for Ageing and Vitality, Newcastle University, Newcastle upon Tyne, NE4 5PL UK; 28 Cardiff University Brain Research Imaging Centre (CUBRIC), Cardiff University, Maindy Road, Cardiff, CF24 4HQ UK

**Keywords:** Dementia, Alzheimer disease, Cholinesterase inhibitors, Muscarinic antagonists, Antidepressive agents, Antipsychotic agents, Cognitive dysfunction, Memory disorders, Cognition, Polypharmacy

## Abstract

**Background:**

Anticholinergic medications are drugs that block cholinergic transmission, either as their primary therapeutic action or as a secondary effect. Patients with dementia may be particularly sensitive to the central effects of anticholinergic drugs. Anticholinergics also antagonise the effects of the main dementia treatment, cholinesterase inhibitors. Our study aimed to investigate anticholinergic prescribing for dementia patients in UK acute hospitals before and after admission.

**Methods:**

We included 352 patients with dementia from 17 UK hospital sites in 2019. They were all inpatients on surgical, medical or Care of the Elderly wards. Information about each patient’s medications were collected using a standardised form, and the anticholinergic drug burden of each patient was calculated with an evidence-based online calculator. Wilcoxon’s rank test was used to look at the correlation between two subgroups upon admission and discharge.

**Results:**

On admission to hospital, 37.8% of patients had an anticholinergic burden score ≥ 1 and 5.68% ≥3. On discharge, 43.2% of patients with an anticholinergic burden score ≥ 1 and 9.1% ≥3. The increase in scores was statistically significant (*p* = 0.001). Psychotropics were the most common group of anticholinergic medications prescribed at discharge. Of those patients taking cholinesterase inhibitors, 44.9% were also prescribed anticholinergic medications.

**Conclusions:**

Our cross-sectional, multicentre study found that people with dementia are commonly prescribed anticholinergic medications, even if concurrently taking cholinesterase inhibitors, and are significantly more likely to be discharged from hospital with a higher anticholinergic burden than on admission.

**Supplementary Information:**

The online version contains supplementary material available at 10.1186/s12877-022-03235-9.

## Background

Dementia is a collective term for chronic neurodegenerative illnesses characterised by progressive cognitive deficits and functional decline [1], of which Alzheimer’s dementia is the most common [2]. The neurotransmitter Acetylcholine is thought to play a key role in memory, and loss of cholinergic neurons correlates with memory loss in Alzheimer’s and other dementias [3].

Anticholinergic medications are medications that block cholinergic transmission, either as their primary therapeutic action or as a secondary effect [4]. Older patients are particularly prone to side-effects from these drugs including impaired memory and attention [5], delirium [6], falls [5], constipation and urinary retention [7]. The effects on cognition seem to be cumulative [8], with increased anticholinergic activity associated with a marked decline in cognition. Patients with dementia may be particularly sensitive to the central effects of anticholinergic drugs. They are more likely to develop delirium and increased cognitive decline [9], potentially due to increased blood-brain barrier permeability [10]. Anticholinergics also antagonise the effects of the main dementia treatment, cholinesterase inhibitors [11]. Examples of such anticholinergic drugs include tricyclic antidepressants, bladder antimuscarinics and antipsychotics [12].

Despite this, older people with dementia are subject to considerable polypharmacy, including drugs with anticholinergic effects [13]. Polypharmacy as a whole [14], and anticholinergic polypharmacy specifically [15], have been correlated with increased hospitalisation and mortality.

Recent studies have attempted to quantify the anticholinergic effect of different medications. Online calculators such as medichec and acbcalc have been developed using these models. Medichec, based on Bishara et al. [16], reports an anticholinergic burden (ACB) score for each drug, calculated using the following factors: i) the magnitude of anticholinergic action, ii) the extent of blood-brain-barrier penetration, and iii) reports of association with cognitive impairment. It also provides an aggregate ACB score based on all drugs prescribed to an individual.

NICE guidelines on the assessment and management of dementia recommend reviewing and, where possible, replacing anticholinergic drugs in patients with dementia [17]. However, it is not clear how often this occurs in clinical practice. Our study aimed to characterise anticholinergic prescribing for dementia patients in UK acute hospitals before and after admission.

## Methods

### Study aim

To characterise anticholinergic prescribing for dementia patients in UK acute hospitals before and after admission.

### Study design

A cross-sectional, multicentre study was conducted in 2019.

### Study sites

Seventeen hospital sites from across the United Kingdom were involved in the study (Additional file 1: Appendix B). To be included in the audit, each hospital site was required to have a minimum of one surgical, one medical and one Care of the Elderly ward. The audit was registered at each site and approval from each trust audit committee was gained prior to data collection.

### Study population

The sample comprised 352 medical records of patients with dementia. They were all inpatients on a surgical, medical or Care of the Elderly wards. Only patients with a formal diagnosis of dementia (pre-existing or made during that admission) were included. We included only patients who were ready for discharge to ensure that we captured their discharge medications.

### Data collection

DC, NE and CG designed a form to capture data on each study participant. The form included: patient demographics, medications on admission and discharge, and discharge destination (Additional file 1: Appendix C). No identifying information was recorded.

Data was collected from February to May 2019. For each participant identified, their medical notes and drug charts were reviewed. The data collection tool Enketo [18] was used to record the data. To calculate the ACB for each participant, an online calculator was used (http://medichec.com [19]). This gave an overall ACB score for each patient based on their list of medications as reported by Bishara et al. [16].

### Data analysis

Data was analysed by AH and DC using the statistical software package ‘R’ [20]. A mean overall ACB Score was calculated for admission and discharge. As the data was non-parametric, Wilcoxon’s rank test was used to ascertain whether this change in score was statistically significant.

Frequencies were calculated to show the number of medications per drug class within the dataset. These were weighted by the effect of the drug and the frequency of prescription to show the contribution to the overall ACB score.

Further analysis looked at the subset of patients taking the anti-dementia cholinesterase inhibitors. The frequency of patients prescribed both cholinesterase inhibitors and ACB medications were calculated, and their change in ACB score from admission to discharge.

Data was recorded on the input patients received from old-age psychiatrists, geriatricians and dementia specialists. Using change in ACB score as the outcome variable, we used linear regression using the ordinary least squares method against the binary input variables of ‘input from each specialty’, modelled as interacting variables. A separate linear regression was performed according to the type of ward the patient was on– modelled as non-interacting binary variables, with change in ACB score as the outcome variable.

### Ethics

As our study was accessing routinely-collected data to evaluate service provision through the collection of anonymised and aggregated data, we registered with local audit committees but did not apply for research ethics. All methods were performed in accordance with the relevant standards for service evaluations.

## Results

### Patient demographics

Three hundred fifty-two patient records were included in the audit across 17 sites. A summary of patient demographics is presented in Table 1.

**Table 1:** Patient demographics

Charactersitic	N	Percentage (%)
**Age (years)**		
< 65	6	1.7
65–74	25	7.1
75–84	137	38.9
85–94	161	45.7
> 95	22	6.3
**Sex**		
Female	190	54
**Dementia Subtype**		
Alzheimer’s	130	36.9
Vascular	80	22.7
Mixed	47	13.4
Lewy Body	34	9.7
Frontotemporal	5	1.4
Other (e.g. Unspecified Dementia, Dementia in Parkinson’s etc)	56	15.9
**Diagnoses of Dementia made during admission**		
Yes	26	7.4
**New diagnoses of dementia made despite delerium recorded during the admission**		
Yes	8	30.1
**Ward**		
Acute	65	18.5
Dementia	34	9.7
Geriatric	186	52.8
Surgical	36	10.2
Other (e.g. Delayed dishcarged ward, Medical rehabilitation etc)	30	8.5
**Specialist Input**		
Geriatrician	216	61.4
Dementia Specialist	18	5.1
Old Age Psychiatirst	4	1.1
Input from 2+ of above	65	18.5
**Admitted from**		
Own Home	265	75.3
Residential Home	41	11.6
Nursing Home	44	12.5
**Discharged to**		
Own Home	148	42.0
Residential Home	74	21.0
Nursing Home	98	27.8
Rehabilitation	24	6.8

Patients were distributed across the hospitals with 18.5% admitted to acute wards, 9.7% admitted to a dementia ward, 52.8% admitted to a geriatrics ward, 10.2% admitted to surgical wards and 8.5% admitted to ‘other’ wards. 86.1% of patients were either seen by a geriatrician, dementia specialist or old age psychiatrist during their admission.

### Anticholinergic burden

**Fig. 1:**
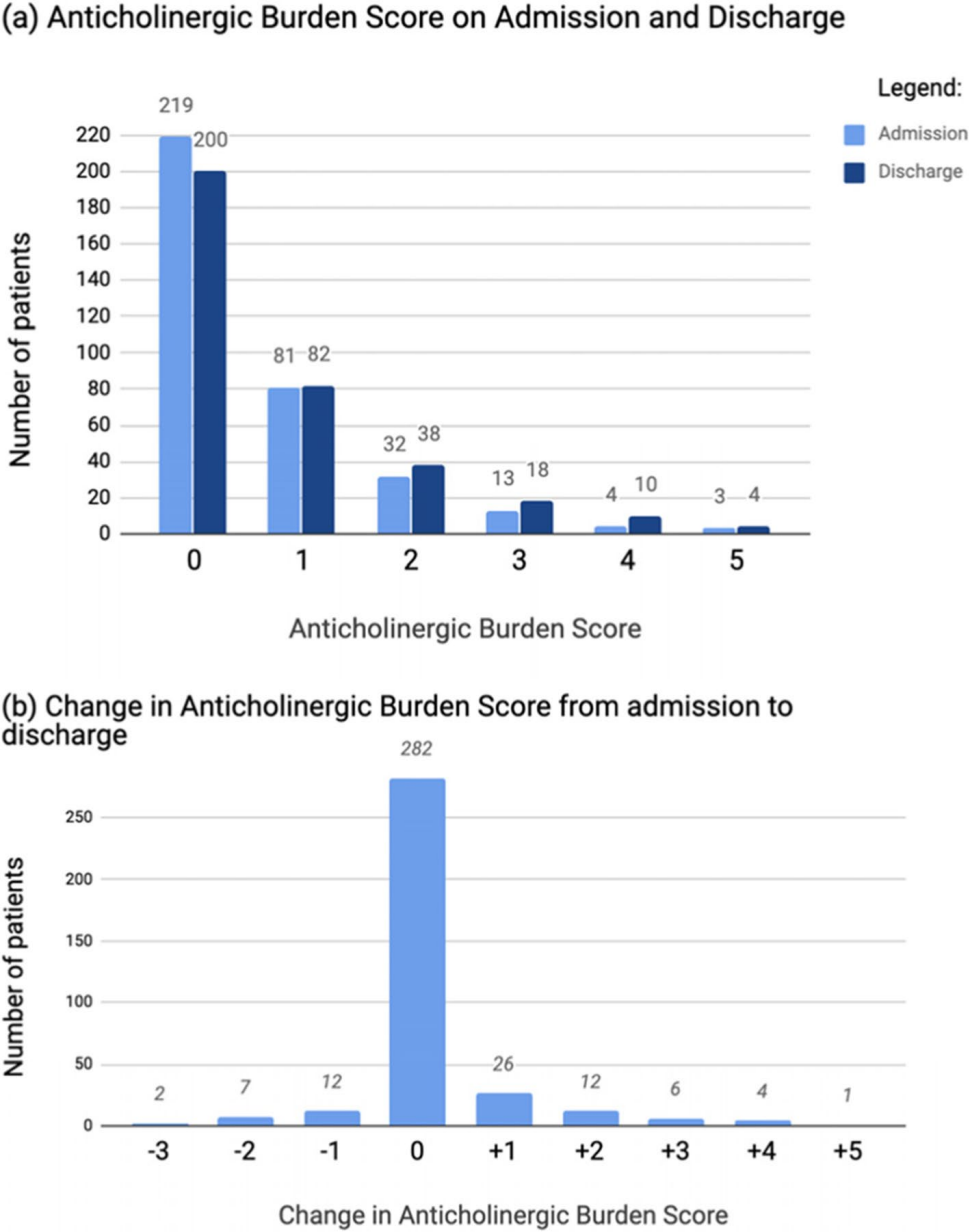
Depicts bar charts (**a**) and (**b**). Chart (**a**) displays the number of patients with each Anticholinergic Burden Score, first at Admission, then at discharge. Chart (**b**) displays the change in Anticholinergic Burden Score from admission to discharge. Negative change shows a decrease in score from admission to discharge. Positive change shows an increase in score from admission to discharge

Figure 1 shows the distribution of ACB scores on admission and on discharge. Mean ACB score on admission was 0.61, with a mean score on discharge of 0.77.

On admission to hospital, 37.8% of patients had an ACB ≥1 and 5.68% had an ACB of ≥3. This reflected a total sample ACB score on admission of 215. On discharge, 43.2% of patients with an ACB score ≥ 1 and 9.1% of patients had an ACB score of ≥3. This corresponded to a total sample ACB of 272. As the value counts for total ACB at admission and total ACB at discharge are non-parametric, Wilcoxon analysis could then be used to compare whether the change from total admission ACB score to total discharge ACB score was significant. This analysis showed that there was in fact a statistically significant increase from total admission ACB score to the total discharge ACB score (t = 693.0, *p* = 0.001).

Table 2 shows the results of regression with change in ACB as the outcome variable and specialist input as the input variables. Significant values included negative association between ACB scores and input from old age psychiatry alone (*p* = 0.024), and from all 3 specialties (*p* = 0.005). Input from both old age psychiatrist and dementia specialist was positively associated with ACB scores (*p* = 0.013).

**Table 2:** Results of regression of specialist input against change in ACB score

Specialist input	***P*** =	0.025	0.975
Geriatrician	0.448	−0.366	0.162
Old age Psychiatry	0.024	−1.867	−0.133
Geriatrician + Old age Psychiatry	0.060	−0.041	1.937
Dementia Specialist	0.476	−0.626	0.293
Geriatrician + Dementia Specialist	0.521	−0.743	0.377
Old Age Psychiatry + Dementia Specialist	0.013	0.311	2.594
Geriatrician + Old age Psychiatry + Dementia Specialist	0.005	−3.297	−0.601

Results of the regression of change in ACB against ward type are shown in Table 3. All wards were significantly positively associated with change in ACB scores other than dementia wards.

**Table 3:** Linear regression analysis testing whether ward type influence the total ACB at discharge

Ward type	***P*** =	0.05	0.95
Acute	0.023	0.283	3.717
Geriatric	0.038	0.104	3.52
Surgical	0.038	0.106	3.56
Dementia	0.057	−0.052	3.405
Other	0.032	0.168	3.632

On discharge, 152 patients were taking anticholinergic medications. 103 (67.8%) were taking one anticholinergic medication, 34 (22.4%) patients were taking two anticholinergic medications, 12 (7.9%) patients were taking three anticholinergic medications and 3 (2.0%) patients were taking four anticholinergic medications. In total, there were 219 instances of anticholinergic medications being prescribed.

Figure 1 shows the distributions of ACB scores at the beginning and end of the admission, as well as the change across the admission.

**Fig. 2:**
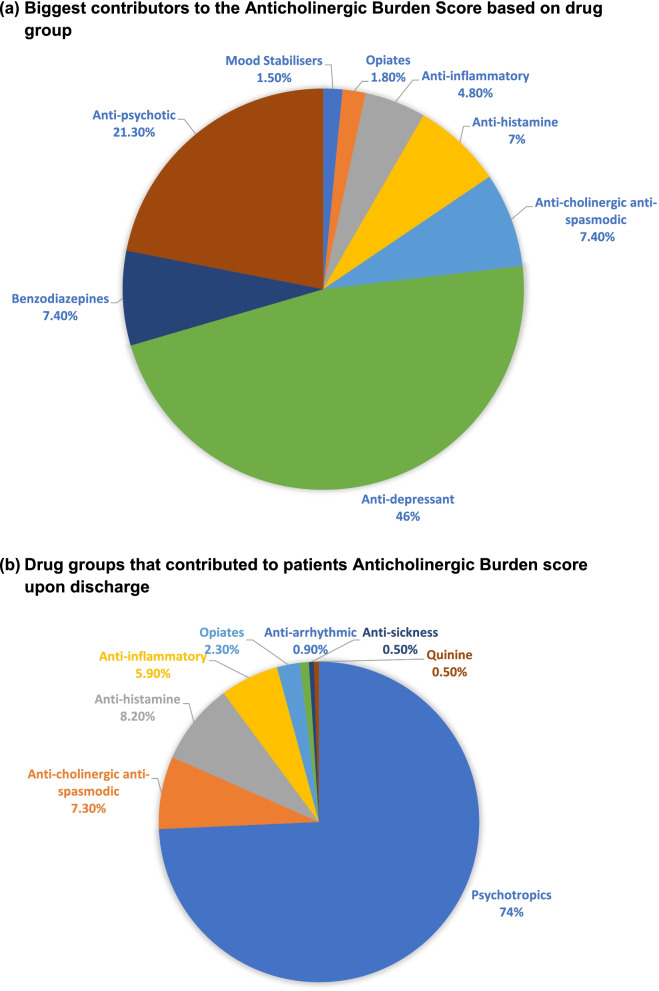
Depicts pie charts (**a**) and (**b**). Chart (**a**) highlights the proportional contributions to the Anticholinergic Burden Score for each drug group. The contribution each drug group makes is based on the total Anticholinergic Burden score, the individual drug Anticholinergic Burden score and its frequency in the dataset. Chart (**b**) depicts the frequency each drug group contributed to the patients Anticholinergic Burden score at discharge

Of the 219 anticholinergic medications prescribed at discharge, the most common drug class was psychotropics (antidepressants, antipsychotics, mood stabilisers or benzodiazepines; 74%) (see Fig. 2). The other drug classes were antihistamines (8.2%), anticholinergic antispasmodics (7.3%), non-steroidal anti-inflammatories (5.9%), opiates (2.3%), anti-arrhythmics, anti-sickness, quinine and Parkinson medications (1.4%). The most frequently prescribed medications with anticholinergic activity were Mirtazapine (*n* = 41), Sertraline (*n* = 35), Citalopram (*n* = 18), Cyclizine (*n* = 17) and Midazolam (*n* = 14). When adjusted for their respective ACB scores, the drugs contributing most to the total ACB were Mirtazapine, Sertraline and Amitriptyline. The breakdown drug classes and their contribution to the ACB scores is shown in Table 4.

**Table 4:** Drugs prescribed, N prescribed, ACB drug score for drug, percentage of total ACB at discharge

Drug Class	Medicine	ACB score	N Prescribed	Percentage of total ACB for sample (%)
Antidepressants		1.4	107	55.07
	Mirtazapine	1	41	15.07
	Sertraline	1	35	12.87
	Amitriptyline	3	9	9.93
	Citalopram	1	18	6.62
	Fluoxetine	1	4	1.47
Antipsychotics		1.67	31	19
	Quetiapine	2	11	8.09
	Olanzapine	2	10	7.35
	Prochlorperazine	2	3	2.21
	Levomepromazine	2	3	2.21
	Aripiprazole	1	3	1.1
	Flupentixol Decanoate	1	1	0.37
Antihistamines		1.5	18	9.93
	Cyclizine	1	17	6.25
	Chlorphenamine	2	1	0.74
Antispasmodics		1.33	16	7.84
	Hyoscine Butyl Bromide	1	9	3.31
	Tolterodine	2	4	2.94
	Solifenacin	1	3	1.1
Benzodiazepines		1	20	7.35
	Midazolam	1	14	5.15
	Diazepam	1	5	1.84
	Temazepam	1	1	0.37
Anti-inflammatories		1	13	4.78
	Prednisolone	1	13	4.78
Opiates		1	5	1.84
	Fentanyl	1	5	1.84
Mood Stabilisers		1	4	1.47
	Carbamazepine	1	3	1.1
	Lithium	1	1	0.37
Anti-sickness		3	1	1.1
	Hyoscine Hydrobromide	3	1	1.1
Anti-arrhythmics		1	2	0.74
	Amiodarone	1	2	0.74
Anti-Parkinson’s		2	1	0.74
	Amantadine	2	1	0.74
Quinine		1	1	0.37
	Quinine Sulphate	1	1	0.37

Seventy-six medications with anticholinergic activity were started during hospital admissions. The most frequently commenced group of medications were psychotropics (63.2%). Of the started medications, 26.3% were antidepressants, 22.4% were antipsychotics, 17.1% were antihistamines, 10.5% were antispasmodics, 14.5% were benzodiazepines, 6.6% were anti-inflammatories and 2.6% were opiates.

Twenty-nine anticholinergic agents were stopped. 69% of stopped anticholinergic medications were psychotropics. The most commonly stopped drug classes were antidepressants (34.5%), followed by antipsychotics (17.2%). Others included antihistamines (10.3%), anticholinergic antispasmodic (10.3%), benzodiazepines (10.3%), anti-inflammatory (6.9%) and mood stabilisers, antiarrhythmic and Parkinson’s medications (3.5%). Table 5 shows the changes in antipsychotic prescribing between admission and discharge.

**Table 5:** Antipsychotics present at discharge (new Vs old Vs change in prescription)

Antipsychotics	N	Percentage (%)
Admitted and discharged on the same antipsychotic	13	41.9
Aripiprazole	1	
Quetiapine	4	
Olanzapine	5	
Levopromazine	2	
Prochlorperazine	0	
Flupentixol	1	
Newly prescribed an antipsychotic (no previous)	17	54.8
Aripiprazole	2	
Quetiapine	7	
Olanzapine	4	
Levopromazine	1	
Prochlorperazine	3	
Change in type of antipsychotic during admission	1	3.2
Risperidone**➔** Olanzapine	1	

Sixty-nine patients were taking anti-dementia cholinesterase inhibitors. Of those patients, 31 (44.9%) were also taking anticholinergic medications. Eighteen patients had an ACB score of 1, eight patients scored 2, three patients scored 4 and one patient scored 5. Two patients on cholinesterase inhibitors had a decreased ACB from admission to discharge. The medications stopped during admission were antipsychotics (100%). Nine patients had an increase in score from admission to discharge. Medications added during admission were antidepressants (20%), antipsychotics (40%), benzodiazepines (20%), anti-inflammatories (10%) and antihistamines (10%). The remaining 58 patients had no change in score between admission and discharge.

## Discussion

Our cross-sectional, multicentre study found that people with dementia are commonly prescribed anticholinergic medications, even if concurrently taking cholinesterase inhibitors, and are significantly more likely to be discharged from hospital with a higher ACB than on admission. We found that psychotropic medications accounted for almost three quarters of anticholinergic medications taken on discharge. Psychotropics are both the most added and the most stopped medication during hospital admissions.

Whilst there is a significant literature on ACB [21, 22], particularly in dementia [23–26], our study adds a large cohort of patients from a variety of hospitals around the UK, with granular patient-level data including treatment setting and specialist input.

Our findings are broadly consistent with the wider literature. Gutierrez-Valencia et al. [21] reported an increase in medications with anticholinergic effects among 200 patients discharged from an acute geriatric unit. Similarly, Wawruch et al. [22] found a statistically significant increase in elderly patients prescribed anticholinergic medications at discharge in 1636 patients admitted to long-term care facilities in Slovakia [22]. In a large retrospective study of Italian dementia patients, Reinold et al. [24] found that ACB was higher at discharge, with 46.1% of patients having a moderate to high ACB score at discharge compared to 25.4% at admission. Of prescribed medications with anticholinergic activity, they reported cardiovascular drugs were the most common, with antipsychotics second. Upon discharge, they noted that furosemide (23.4%), quetiapine (15.3%) and promazine (8.9%) were most commonly prescribed [24].

Whilst we also found an increased ACB at discharge and identified antipsychotics as the second biggest malefactor (22.4% of anticholinergic medications), in our study antidepressants were the largest contributor to ACB score (26.3%). The three most commonly prescribed medications were mirtazapine, sertraline and citalopram, and the drugs contributing most to the total ACB were mirtazapine, sertraline and amitriptyline. Psychotropic medications are frequently started in acute hospitals. In an audit of 35 acute hospitals in Ireland in 2013, Gallagher et al. [23] reported a significant increase in patients prescribed antipsychotics on discharge [23]. Indeed, 41% of those prescribed antipsychotics had doses increased [23].

Almost half of patients in our study (44.9%) who were prescribed acetylcholinesterase inhibitors as treatment for dementia were also given anticholinergic drugs. A small number of these patients (< 3%) had anticholinergic medications stopped or reduced. Other studies have also reported that inappropriate co-prescription of anticholinergics and acetylcholinesterase inhibitors is common [11]. The action of acetylcholinesterase inhibitors is opposed by anticholinergic medications [27], and this may lead to a reduction in therapeutic benefit.

In our study, the majority of patients (86.1%) were seen either by a geriatrician, dementia specialist or old age psychiatrist during their admission. Our analysis shows that specialist input was not associated with increased ACB, and that input from old-age psychiatry and a combination of old age psychiatry, geriatrician and dementia specialist was associated with a reduction in ACB, although this finding must be treated with significant caution due to the small numbers (*n* = 38 and 14 respectively) involved. It was also notable that, of all wards, only dementia wards were not associated with increases in anticholinergic burden. This presents some evidence that specialist input may be helpful in counteracting the tendency towards increasing anticholinergic burden in inpatients. This is despite recommendations that reviews can be undertaken by any doctor or pharmacist or specialist nurse [28, 29], with the use of tools such as STOPP/START Version 2 (Screening Tool of Older People’s Prescriptions and Screening Tool to Alert to Right Treatment) [30].

Although there is consensus on many findings, it is important to note the heterogeneity in methodology. Each study uses a different method to assess ACB. For example, Wawruch et al. [22] identified anticholinergic drugs as those that scored ≥2 of 3 on lists published by Han et al. [8] and Rudolph et al. [31], but did not calculate an ACB score for individual patients. Gutierrez-Valencia et al. [21] used the anticholinergic risk scale (ARS) based on Rudolph et al. [31] The ARS ranks medications known to have anticholinergic effects on a scale from 0 (limited/none) to 3 (very strong). This ranking is based on the medication’s dissociation constant for the cholinergic receptor and its anticholinergic adverse effects. A patient’s ARS score is the sum of the ARS ranking for all of their medications [31]. However, the ARS is not based on a systematic review of the literature and instead identifies medications based on one American healthcare system. In contrast, Reinold et al. [24] used the ‘Anticholinergic Cognitive Burden Scale’ based on Boustani et al. [30] The developers of this scale classified medications into mild, moderate or severe anticholinergic effects based on a literature review and evaluation by an expert interdisciplinary team. These medications were then scored from 0 to 3 (0 = no anticholinergic effect, 1 = possible anticholinergic effects, 2/3 = clinically relevant anticholinergic effects) based on their in vitro affinity for muscarinic receptors, blood-brain barrier permeability and association with development of delirium. A patient’s total score is the sum of the score assigned to each medication [32]. Another possible system is the Anticholinergic Drug Scale developed by Carnahan et al. [33] This scale ranks medications based on their serum anticholinergic activity from 0 (no known activity) to 3 (marked activity). The total score for each patient is determined by the sum of the score for each medication [31]. In our study we chose to use Bishara et al.’s [16] Anticholinergic Effect on Cognition (AEC) scale, as described above. The AEC scale uses information on drug classes and medications from the British National Formulary. It also assesses penetration of the blood-brain barrier [16].

### Strengths

This study has a number of strengths. We are the first to investigate anti-cholinergic prescribing in dementia patients in UK hospitals. Our study was a cross-sectional study, comprising 17 sites. Therefore, the results should be generalisable to other acute hospital settings. We have a large sample size, which minimised the chance of type II error. We used an evidence-based online calculator to ensure that we made standardised assessments of ACB score.

### Limitations

Some limitations should be noted. We used a convenience sample of dementia patients who were ready for discharge. This could have introduced some selection bias. We did not enhance case ascertainment by using standardised dementia diagnostic assessments. Thus, patients that did not have a diagnosis of dementia made by their clinical team and clearly documented would have been missed. Whilst Bishara et al.’s [16] AEC scale comprehensively assesses adverse effects due to anti-cholinergic action, it does not consider the broader appropriateness of the prescription [19]. Future studies would benefit from longitudinal follow-up of patients after hospital discharge, to assess the effect of anticholinergic prescriptions on long-term outcomes.

## Conclusions

Our cross-sectional, multicentre study found that older adults with dementia are commonly prescribed anticholinergic medications, even if also prescribed cholinesterase inhibitors. Anticholinergic burden increased after hospital admission. Psychotropic medications accounted for the majority of anticholinergic burden. Increased awareness of anticholinergic effects and standardised tools for medication reviews may help to address this problem. It may be that specialist input from old age psychiatry along with other specialists or specialist dementia wards can help reduce this tendency toward increased anticholinergic burden, but this is a preliminary finding that requires further validation. Further research is needed to investigate the impact of anticholinergic burden on long-term patient outcomes.

## Supplementary Information

**Additional file 1: Appendix A.** SPARC collaborators. **Appendix B.** Table 1 - Details of the 17 hospital sites in the United Kingdom involved in the study. **Appendix C.** Data Collection Form (questions included on software Enketo [18]).

## Data Availability

The datasets used and analysed during the current study are available from the corresponding author, AH, on reasonable request.

## Abbreviations

ACB: Anticholinergic burden
STOPP/START Version 2: Screening Tool of Older People’s Prescriptions and Screening Tool to Alert to Right Treatment Version 2
ARS: Anticholinergic risk scale
AEC scale: Anticholinergic Effect on Cognition scale

## References

[1] Alzheimer Society of Canada (2019). Mild cognitive impairment.

[2] Brunnstrom H, Gustafson L, Passant U, Englund E (2009). Prevalence of dementia subtypes: a 30-year retrospective survey of neuropathological reports. Arch Gerontol Geriatr.

[3] Perry E (1986). The cholinergic hypothesis - ten years on. Br Med Bull.

[4] Gerretsen P, Pollock B (2011). Drugs with anticholinergic properties: a current perspective on use and safety. Expert Opin Drug Saf.

[5] Aizenberg D, Sigler M, Weizman A, Barak Y (2002). Anticholinergic burden and the risk of falls among elderly psychiatric inpatients: a 4-year case-control study. Int Psychogeriatr.

[6] Flacker J, Cummings V, Mach J, Bettin K, Kiely D, Wei J (1998). The association of serum anticholinergic activity with delirium in elderly medical patients. Am J Geriatr Psychiatry.

[7] Lieberman J (2004). Managing anticholinergic side effects. Prim Care Companion J Clin Psychiatry.

[8] Han L, Agostini J, Allore H (2008). Cumulative anticholinergic exposure is associated with poor memory and executive function in older men. J Am Geriatr Soc.

[9] Fox C, Richardson K, Maidment I (2011). Anticholingeric medication use and cognitive impairment in the older population: the medical research council cognitive impairment in the older population: the medical research council cognitive function and ageing study. J Am Geriatr Soc.

[10] Campbell N, Boustani M, Limbil T (2009). The cognitive impact of anticholinergics: a clinical review. Clin Interv Aging.

[11] Johnell K, Fastbom J (2008). Concurrent use of anticholinergic drugs and cholinesterase inhibitors: register-based study of over 700,000 elderly patients. Drugs Aging.

[12] Gray S, Hanlon J (2016). Anticholinergic medication use and dementia: latest evidence and clinical implications. Ther Adv Drug Saf.

[13] Sergi G, De Rui M, Sarti S, Manzato E (2011). Polypharmacy in the elderly: can comprehensive geriatric assessment reduce inappropriate medication use?. Drugs Aging.

[14] Mueller C, Molokhia M, Perera G (2018). Polypharmacy in people with dementia: associations with adverse health outcomes. Exp Gerontol.

[15] Bishara D, Perera G, Harwood D, et al. The anticholinergic effect on cognition (AEC) scale - associations with mortality, hospitalisation and cognitive decline following dementia diagnosis. Int J Geriatr Psychiatry. 2020:1–9. 10.1002/gps.5330. 10.1002/gps.5330 32394521

[16] Bishara D, Harwood D, Sauer J, Taylor D (2017). Anticholinergic effect on cognition (AEC) of drugs commonly used in older people. Int J Geriatr Psychiatry.

[17] National Institute for Health and Care Excellence (2020). Dementia: assessment, management and support for people living with dementia and their carer NICE guideline [NG97].

[18] Sharif B, Lundin R, Morgan P (2016). Developing a digital data collection platform to measure the prevalence of sepsis in Wales. J Am Med Inform Assoc.

[19] South London and Maudsley NHS Foundation Trust (2020). The anticholinergic effect on cognition tool.

[20] R Core Team. R: A language and environment for statistical computing. Vienna: R Foundation for Statistical Computing; 2022. https://www.R-project.org/.

[21] Gutierrez-Valencia M, Izquierdo M, Malafarina V (2017). Impact of hospitalization in an acute geriatric unit on polypharmacy and potentially inappropriate prescriptions: a retrospective study. Geriatr Gerontol Int.

[22] Wawruch M, Macugova A, Kostkova L (2012). The use of medications with anticholinergic properties and risk factors for their use in hospitalised elderly patients. Pharmacoepidemiol Drug Saf.

[23] Gallagher P, Curtin D, de Siun A (2016). Antipsychotic prescription amongst hospitalized patients with dementia. QJM.

[24] Reinold J, Palese F, Romanese F, Logroscino G, Riedel O, Pisa FE (2019). Anticholinergic burden before and after hospitalization in older adults with dementia: increase due to antipsychotic medications. Int J Geriatr Psychiatry.

[25] Weichert I, Romero-Ortuno R, Tolonen J, Soe T, Lebus C, Choudhury S, Nadarajah CV, Nanayakkara P, Orrù M, Di Somma S, Global Research on Acute Conditions Team (GREAT) (2018). Anticholinergic medications in patients admitted with cognitive impairment or falls (AMiCI). The impact of hospital admission on anticholinergic cognitive medication burden. Results of a multicentre observational study. J Clin Pharm Ther.

[26] Kable A, Fullerton A, Fraser S (2019). Comparison of potentially inappropriate medications for people with dementia at admission and discharge during an unplanned admission to hospital: results from the SMS dementia study. Healthcare (Basel).

[27] Nair V, Hunter J (2004). Anticholinesterases and anticholinergic drugs. Contin Educ Anaesth Crit Care Pain.

[28] National Institute for Health and Care Excellence (2020). Medicines optimisations Quality Standard [QS120].

[29] National Institute for Health and Care Excellence (2020). Medicines management in care homes Quality standard [QS85].

[30] O'Mahony D, O'Sullivan D, Byrne S, O’Connor M, Ryan C, Gallagher P (2015). STOPP/START criteria for potentially inappropriate prescribing in older people: version 2. Age Ageing.

[31] Rudolph J, Salow M, Angelini M, McGlinchey R (2008). The anticholinergic risk scale and anticholinergic adverse effects in older persons. Arch Intern Med.

[32] Boustani M, Campbell N, Munger S, Maidment I, Fox C (2008). Impact of anticholingerics on the aging brain: a review and practical application. Aging Health.

[33] Carnahan R, Lund B, Perry P, Pollock B, Culp K (2006). The anticholinergic drug scale as a measure of drug-related anticholinergic burden: associations with serum anticholinergic activity. J Clin Pharamacol.

