# Supplementary material for: Anti-cholinergic drug burden in patients with dementia increases after hospital admission: a multicentre cross-sectional study
Source: BMC Geriatr. 2022 Oct 6;22:783. doi: 10.1186/s12877-022-03235-9 (PMC9541078; doi:10.1186/s12877-022-03235-9)
Supplement: Supplementary file 1 — Additional file 1: Appendix A. SPARC collaborators. Appendix B. Table 1 - Details of the 17 hospital sites in the United Kingdom involved in the study. Appendix C. Data Collection Form (questions included on software Enketo [18]). [file 12877_2022_3235_MOESM1_ESM.docx]

**APPENDIX A** – SPARC collaborators

The authors would like to thank the following SPARC collaborators for their help on the project: Aashka Murdeshwar, Abigail Embaie, Ahmad Elmansouri, Alex Gordon, Alison Lerant, Amber-Mai Lewis, Amy Haeffner, Amy Pinkey, Anastasiya Unnikrishnan, Andie Lun, Arina Toma, Arissa James, Ashwin Singh, Azjad Y Elmubarak, Caitlin Culyer, Callum Bruce, Catriona Phillips, Chadek A Rahaman, Chinmay Tijare, Christine Cadman, Claire Hammett, Danish Hafeez, Dengui Zhou, Dottie Duklas, Edward Court, Elena Taylor, Elisa Wylleman, Emilia Jewell, Emilie Dommett, Fatimah Khoda, Freya Cooper, Hannah Egueye, Hans Johnson, Harrison Johal, Hassan Qadri, Helen Rayner, Herkiran Kambo, Holly Thompson, Iain Logan, Iain Williams, Indu Dev, James McLaren, James Russel, Jennifer Appleby, Jenny Humphries, Jess Ye, Jessica Chalmers, Jessica Henry, Jonathan Chan, Jonathan Denfhy, Julia Egierszdorff, Katherine Chin, Katie Turner, Katrina Paton, Katy Brown, Kimberley F Thirlwall, Kirsten Webb, Krishna Ravulapalli, Laetitia Hawkins-Hooker, Laura Sevenoaks, Louise Cox, Lynda Imessaoudene, Manahara Ratnayake, Matthew Butler, Meghan Coyle, Mhairi Reed-Embleton, Monisha Dhar, Nawal Zia, Neil Riley, Niamh Toner, Nigel Gill, Nigin Ghani, Nithya Kadiyala, Orthi Shahzad, Prasanna Rao-Balakrishna, Rahul Shah, Rashid E Reid, Raven Jorgensen, Richard Chater, Richard Telford, Ruchi Chugh, Rukudzo Msindo, Sabari Muthukrishnan, Sadhana Kalidindi, Sagar Shrivastva, Saira Nawaz, Sakshi Dasgupta, Samirah Musasizi, Sapna Patel, Sarah Pendlebury, Sarah Saadat, Seetal Assi, Shammah Gbenga-Ojo, Shanze Ashai, Shruthi Rayen, Simon Manchip, Simran Dass, Sophia Pepes, Sophie Hawkins, Sophie Langford, Sophie Wilson, Soundarya Soundararajan, Sukhbir Khosah, Susmi Suresh, Tafsir Ahmed, Tom Davies, Tom Smith, Usmaan NA Rana, Vashist Motkur, Vinay Mandagere, Viyas Sooriyakumaran, Wen Q Cheok, William Adams, Zak Houghton.

**APPENDIX B** Table 1 - Details of the 17 hospital sites in the United Kingdom involved in the study.

| Area of the United Kingdom | Hospital(s) involved in the study |
| --- | --- |
| Barts | Whipps Cross University Hospital |
| Birmingham | Queen Elizabeth Hospital Birmingham |
| Bristol | Southmead Hospital, Royal United Hospital Bath, Bristol Royal Infirmary, Great Western Hospital |
| Cambridge | Addenbrookes Hospital |
| Durham | James Cook University Hospital |
| Exeter | Royal Devon and Exeter Hospital |
| Keele | Royal Stoke Hospital |
| Lancaster | Royal Lancaster Infirmary, Furness General Hospital |
| Manchester | Manchester Royal Infirmary |
| Newcastle | Sunderland Royal Hospital |
| Norwich | Norfolk and Norwich University Hospital |
| Preston | Royal Preston Hospital |
| Southampton | Southampton General Hospital |
| Oxford | John Radcliffe Hospital |
| Plymouth | Derriford Hospital |
| UCL | Highgate Mental Health Centre |

**APPENDIX C** - Data Collection Form (questions included on software Enketo^18^)

- Patient study number
- Hospital
- Medically fit for discharge
- Sex
- Age
- Admission date
- Reason for admission
- Place of residence before admission
- Reason not yet discharged
- Ward type
- Input from what teams?
- Type of dementia
- Dementia diagnosis made during this admission?
- Delirium present on this admission?
- Total anticholinergic burden (ACB) score on admission
- Total ACB score now
- Is the patient taking cholinesterase inhibitor?
- Which cholinesterase inhibitor?
- Was anticholinergic burden noted in patient notes?
- Was risk of adding anticholinergic medication discussed?
- Reason for medication added
- Was risk of stopping or reducing anticholinergic medication discussed?
- Has follow-up for anticholinergic drugs in the community been suggested?
- Discharge destination
- For each medication:
  - Medication name
  - ACB score for that medication
  - Taking on admission?
  - Dose on admission
  - Taking now?
  - Dose now
